# Inverse photonic design of functional elements that focus Bloch surface waves

**DOI:** 10.1038/s41377-018-0106-x

**Published:** 2018-12-12

**Authors:** Yannick Augenstein, Andreas Vetter, Babak Vosoughi Lahijani, Hans Peter Herzig, Carsten Rockstuhl, Myun-Sik Kim

**Affiliations:** 1 Institute of Theoretical Solid State Physics, Karlsruhe Institute of Technology, 76131 Karlsruhe, Germany; 2 Institute of Nanotechnology, Karlsruhe Institute of Technology, 76344 Eggenstein-Leopoldshafen, Germany; 3 SUSS MicroOptics SA, Rogues-Terres 61, Hauterive, 2068 Switzerland; 4 Optics & Photonics Technology Laboratory, Ecole Polytechnique Fédérale de Lausanne (EPFL), Neuchâtel, Switzerland

## Abstract

Bloch surface waves (BSWs) are sustained at the interface of a suitably designed one-dimensional (1D) dielectric photonic crystal and an ambient material. The elements that control the propagation of BSWs are defined by a spatially structured device layer on top of the 1D photonic crystal that locally changes the effective index of the BSW. An example of such an element is a focusing device that squeezes an incident BSW into a tiny space. However, the ability to focus BSWs is limited since the index contrast achievable with the device layer is usually only on the order of Δ*n*≈0.1 for practical reasons. Conventional elements, e.g., discs or triangles, which rely on a photonic nanojet to focus BSWs, operate insufficiently at such a low index contrast. To solve this problem, we utilize an inverse photonic design strategy to attain functional elements that focus BSWs efficiently into spatial domains slightly smaller than half the wavelength. Selected examples of such functional elements are fabricated. Their ability to focus BSWs is experimentally verified by measuring the field distributions with a scanning near-field optical microscope. Our focusing elements are promising ingredients for a future generation of integrated photonic devices that rely on BSWs, e.g., to carry information, or lab-on-chip devices for specific sensing applications.

## Photonics: Controlling waves on a surface

Light waves confined to travel at the interface between a photonic crystal and an ordinary material, known as Bloch Surface Waves (BSWs), can be focused and controlled in optical circuits by the patterning of an additional surface layer. Controlling BSWs constitutes a challenge but is vital for many applications in information transmission, acquisition, and processing. While traditional approaches based on conventional rational designs only led to a limited performance, researchers from Switzerland and Germany made now use of modern approaches for the computational photonic material design to manipulate BSWs. With suitably designed patterns they could demonstrate the focusing of BSWs down to the diffraction limit; and even below if the BSW was focused close to their element. This ability to focus BSWs unlocks a plethora of immediate applications for novel ‘lab-on-chip’ chemical sensing devices.

## Introduction

The control of electromagnetic fields in integrated environments is of paramount importance for a large number of applications in the broader context of information transmission, acquisition, and processing^1–5^. Traditionally, light in an integrated environment is controlled by waveguides that confine the light in the bulk of some media^6,7^. However, even stronger integration with better accessibility is achieved by confining electromagnetic waves to surfaces^8^. This led to the notion of surface waves, i.e., self-consistent solutions to Maxwell’s equations localized at the interface between two media that exponentially decay away from the interface. Surface waves are characterized by a field profile in the direction normal to the interface and a dispersion relation that governs their propagation along the interface.

The most prominent surface waves are potentially surface plasmon polaritons (SPPs). SPPs are sustained at the interface between a metal and a dielectric. SPPs consist of a hybrid excitation in which some fraction of the energy is stored in the electromagnetic field while another fraction is stored in charge density oscillations of the conduction electrons in the metal^9^. While exploiting the coupling of light to an electronic excitation, the concentration of electromagnetic fields in a nanometric region close to the interface can be achieved. This has been instrumental for a large number of applications, e.g., to sense molecules or, more generally, to guide light at small length scales^10^. However, there is no free lunch; the large confinement of the fields in the metal also results in the dissipation of SPPs. This limits their propagation lengths. Typically, propagation lengths on the order of microns or at most tens of microns are achieved^9^. This rather short propagation length scale is considered to be an obstacle for many applications, and alternative surface waves that do not suffer from this limitation are explored.

The most appealing solution relies on Bloch surface waves (BSWs). BSWs are confined at the interface between an ambient material, i.e., air in our case, and a dielectric one-dimensional (1D) photonic crystal (PC)^11^. The PC consists of an alternating sequence of low- and high-permittivity dielectric materials. BSWs reside in the spectral region of the photonic band gap of the PC. They possess an evanescent field that decays exponentially in both the air medium and the PC and propagate along the interface of the PC. Propagation lengths on the order of millimeters for BSWs designed to operate at near-infrared wavelengths have been reported^12^. The propagation lengths, in the limit of vanishing intrinsic material absorption and the absence of scattering losses, ultimately are only limited by the number of layers in the PC. Such appealing characteristics make BSWs suitable for many sensing applications^13–15^ and also for enhancing the interaction with a magnetic optical field^5^ and supporting organic polaritons that result from the strong coupling between a BSW and organic excitons^16^.

To control the propagation of BSWs, a spatially structured dielectric device layer is usually deposited on top of the PC. The device layer modifies the dispersion relation of the BSW locally and changes the effective index experienced by the BSW^17^. As such, trajectories for light propagation can be defined, and multiple functional elements thereof have been investigated in the past. In particular, elements have been investigated that enable the BSW to impinge on a tiny spatial domain using the photonic nanojet effect^18^. This ability is particularly crucial for future application perspectives in the broader field of lab-on-chip sensing devices^19,20^. There, a fluid containing a substance to be detected flows through a channel, and the fluid should ideally interact with the BSW in a spatial domain as small as possible.

However, in most material platforms for BSWs, the accessible index contrast resulting from the propagation constant of the BSW in the absence/presence of the device layer on top of the 1D PC is rather small, i.e., on the order of Δ*n*≈0.1. A larger index contrast is possible in theory^21^ but because of practical difficulties has not been demonstrated. The achievable low index contrast is insufficient for a basic element, such as a circular disc, to efficiently focus the incident BSW. Other geometries have been investigated based on the rational design of selected geometries, and only recently, isosceles triangles have been identified as the most suitable geometry^18^. Subwavelength focusing was possible, but due to the limited index contrast, the focal width was well above half the wavelength, which is typically considered to be a lower bound for the focal width of far-field optical devices. Additionally, the field amplitude in the focus was rather small, and the undesirable side lobes were well pronounced.

To mitigate these limitations, here we rely on a computational strategy for inverse photonic design to identify elements that can focus BSWs more strongly than elements perceived by rational design. Our approach delivers material distributions on a checkerboard that focus the BSWs to a width nearly exactly half the wavelength of the BSW. We demonstrate the impact of increasing spatial resolution in the definition of the checkerboard pattern on the ability to better focus the incident BSW. We even obtain a focal width that is slightly smaller than half the wavelength when the focal point is placed directly behind the element. This is realized by exploiting near-field components directly behind the structured device. Selected devices are fabricated, and we experimentally demonstrate their anticipated functionality by means of measurements with a scanning near-field optical microscope (SNOM)^22^. All together, we demonstrate that computational strategies for inverse photonic design are suitable to achieve functional elements that can control the propagation of BSWs to an extraordinary degree. The complex character of the structure allows for improved performance with respect to devices designed by a rational approach and constitutes an important step for the integration of BSWs for applications. Our approach can be used for other surface waves in which the limited index contrast imposes limitations in the design of functional photonic elements.

## Results

### Design

**Fig. 1:**
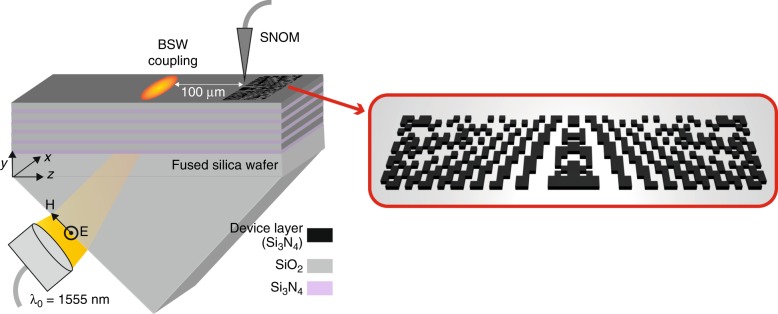
Schematic of the experimental scheme (left) and an illustration of a relevant functional element (right). A 1D PC supports the BSW. The BSW is excited by means of frustrated total internal reflection at a free space wavelength of 1555 nm with TE polarization. The intensity distribution around the functional elements is imaged by a SNOM

The anticipated device is sketched in Fig. 1 along with the experimental prism coupling setup. It consists of the 1D PC on top of a glass prism. The PC is decorated with a spatially structured device layer that defines the functionality by means of refractive index contrast. BSWs are launched by frustrated total internal reflection in a spatial region sufficiently ahead of the functional element, i.e., on the order of 100 μm. The functional element, as designed by computational means, is shown in more detail in the inset of Fig. 1. It consists of a spatial domain in which the device layer is structured in a checkerboard pattern. In the examples presented in this manuscript, the spatial extent of the functional element is 40 μm in the *x*-direction, i.e., normal to the propagation direction of the BSW, and 10 μm in the *z*-direction, i.e., parallel to the propagation direction of the BSW.

The spatial resolution of the checkerboard structure is subject to modifications. Once the BSW is launched, it propagates towards the functional element. The functional element focuses the BSW as strongly as possible in a predefined distance behind the terminating edge of the element. As indicated in Fig. 1, the functionality is verified by measuring the intensity distribution of the BSW using a SNOM. The design of our optical chip consists of two steps. First, we must define the properties of the platform that sustains the BSWs. Second, we have to design the spatial distribution of the device layer that focuses the BSW in a predefined spatial region.

For the first part we rely on an established platform. We consider a fused silica substrate on top of which five double layers of Si_3_N_4_ and SiO_2_ are deposited to define the 1D PC. The top device layer is made from Si_3_N_4_. The free space wavelength of the device is *λ*_0_=1555 nm. The layer stack is designed to sustain a BSW with TE polarization, and an extension of our results to TM polarization is feasible^23,24^. The effective index without/with the device layer amounts to $n_{{\mathrm{eff}}}^{{\mathrm{w}}/{\mathrm{o}}} = 1.1$ and $n_{{\mathrm{eff}}}^{\mathrm{w}} = 1.2$, and this defines the wavelength of the BSW $\lambda _{{\mathrm{BSW}}} = \lambda _0/n_{{\mathrm{eff}}}^{{\mathrm{w}}/{\mathrm{o}}}$. More details on the multilayer design and dispersion curves are documented in the Materials and Methods Section and the Supplementary Information.

To design the functional elements, our objective is to maximize the electric field intensity within a region *χ* representing the focal spot at some distance *z* behind the functional element. As we have no prior information about the achievable focal width, it stands to reason that optimizing the intensity over a small region of space will naturally lead to the formation of a tighter focal point. The optimization problem can be stated as:

1 $$\mathop {{\max }}\limits_\psi \mathop {\smallint }\limits_{a_z}^{b_z} \mathop {\smallint }\limits_{a_x}^{b_x} {\mathrm {d}}x{\mathrm {d}}z\left( {\left| {E_{\mathrm{x}}\left( {x,z} \right)} \right|^2 + \left| {E_{\mathrm{z}}\left( {x,z} \right)} \right|^2} \right)$$

where *ψ* represents the material distribution in the design region. [*a*,*b*] denotes the interval over which the localization of the focus is restricted. Having specified the design region, objective region, and focal distance of the device (see Materials and Methods section), we only need to define a resolution for the material grid, i.e., the feature size of the dielectric inclusions. Then, the material at each grid point needs to be optimized. The optimization method, which is inspired by the literature^25^, proceeds as follows. From an initial geometry, we test a separate inclusion at each location of the material grid and keep the one that maximally improves our design objective. In each iteration of the optimization, the impact of modifying each possible inclusion on the figure of merit (FOM) is simulated in 2D (see Materials and Methods section for details on the simulation method). The FOM is calculated according to the objective of maximizing the intensity in the focal spot. The modification of the inclusion that shows the largest improvement in the FOM is kept, while the others are discarded, and the next iteration begins. In case none of the inclusions leads to an improvement in the FOM, the algorithm uses a suboptimal inclusion and continues with the next iteration. Should the FOM improve in the next iteration, the optimization continues normally. If it does not, the algorithm considers another suboptimal inclusion in its search. The depth of this suboptimal search procedure is controlled by a parameter in the optimization routine. Generally, a shallower search leads to faster convergence but worse end results, and conversely, a deeper search leads to slower convergence but better results. We found a search depth of 3 (number of suboptimal inclusions allowed) to be suited for our problem. This somewhat exhaustive search mitigates the problem of rapid convergence into local optima while still guaranteeing convergence over time. The optimization landscape of our problem contains many local minima with different material distributions but similar performance. Our algorithm does not aim to find a global optimum for the problem but rather a local minimum that meets our design targets (e.g., regarding focal width and field intensity). Further aspects of the optimization are discussed in the Materials and Methods section and in the Supplementary Information.

Taking all these aspects together, we can design functional elements that occupy a given spatial domain in which the material is distributed according to predefined constraints. The purpose of the elements is to focus the incident BSW into the smallest spatial region at a certain working distance behind the terminating edge of the functional element.

**Fig. 2:**
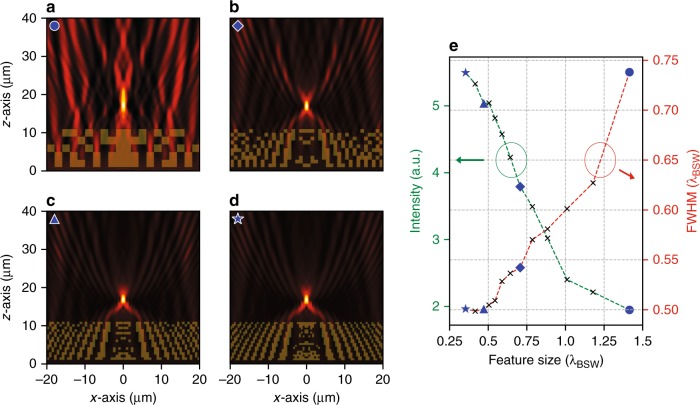
Optimization of different structures that can focus BSWs at some distance behind the functional element, i.e., 5 µm in the present case. On the left (**a**–**d**), we show the electric field intensity distributions upon illuminating optimized elements with a plane wave for selected spatial discretization of the structures. The optimized structures are shown as an overlay. The considered grid size corresponds to **a** 2 μm (=1.414 λ_BSW_), **b** 1 μm (=0.707 λ_BSW_), **c** 667 nm (=0.471 λ_BSW_), and **d** 500 nm (=0.354 λ_BSW_), respectively. **e** We show the achievable focal width and peak intensity at the focal point of the elements as a function of the feature size of the grid. Selected data points correspond to the samples shown in **a**–**d** as marked by the symbol in the top left corner of each intensity distribution

Selected results of the above design involving Eq. (1) are shown in Fig. 2, where we demonstrate the ability of a functional element to focus the incident BSW at some distance behind the terminating edge of the functional element, i.e., 5 µm in this case. In this simulation, we made a sequence of optimizations by varying the number of pixels to discretize the functional elements. This translates to a feature size of our grid with which the space is discretized. The intensity distributions of the electric field of selected designs are shown in Fig. 2a–d, where the feature sizes of the grid are 2μm ( = 1.414 λ_BSW_), 1μm ( = 0.707 λ_BSW_), 667 nm ( = 0.471 λ_BSW_), and 500 nm ( = 0.354 λ_BSW_), respectively. In Fig. 2e, we show the peak intensity of the focal spot and the focal width from the intensities shown in Fig. 2a–d along with some more data points. The focal width was measured as the full-width-at-half-maximum (FWHM) of the intensity in the focus. By means of 2D simulations, we demonstrate that the devices are rather robust against changes in the illumination profile, the angle of incidence, and the operation wavelength. These findings are reported in the Supplementary Information (see Section 7).

It is clear that a tighter focus can be achieved for a smaller feature size resolution. This result is not surprising, as more degrees of freedom are provided that affect the propagation of the BSW. One can also notice that decreasing the spatial resolution below a certain level (e.g., approximately smaller than 0.5 λ_BSW_) does not necessarily improve the foci quality. That makes sense because the focal width is fundamentally bound by the resolution limit, and upon approaching it, further improvement is prohibited. In other words, if the structuring takes place on lengths much smaller than the wavelength of the BSW, the BSW does not resolve the spatial details of the structure anymore but experiences an effective medium instead. The only thing that can be further improved with a refined spatial distribution is the maximal field intensity of the focus. This is possible due to the suppression of side lobes in the field distribution behind the functional element that allows squeezing more of the incident energy into the focal region.

While contemplating the computationally optimized structures, a few insights can be obtained as to why the computer found them. Obviously, there is a tendency in the elements to consist of rather thin and lengthy waveguiding strings. They collect the BSW from an extended region and funnel it into a tiny central space at the upper part of the element. The phase accumulation of the BSW propagating in each of these strings on both sides with respect to the optical axis must be matched to result in constructive interference in the center. This allows efficient focusing of the BSWs. Although many details of the structure are hard to explain, they serve the anticipated purpose. We see this not only in the reduced focal width but also in the increased intensity/energy contained in the central spot. The ability of the designed elements to focus BSWs is superior when compared to the elements obtained by a rational design approach^18^. We stress that the samples presented here are only exemplary. Elements with comparable functionality and different design constraints have also been identified. To verify the suitability of our approach by experiments, we fabricated selected elements and characterized them by means of a SNOM. The results of this study are presented below.

### Device characterization

Here, we focus on two different devices to demonstrate our concept. The first device is one from the simulations that have been discussed in the previous section. To be precise, we considered the device with a grid size of 667 nm that focuses the incident BSW at a distance of 5 µm behind the edge of the element and produces a focal width that corresponds to half the wavelength of the BSW. This corresponds to the optimized design result shown in Fig. 2c. To go beyond the fundamental half-wavelength barrier for the focal width, we also study a second device designed to focus the BSW directly behind the terminating edge of the functional element. As a result, we capitalize on near-field effects (with respect to the distance of the focus from the device edge) to focus the BSW into an even smaller region. Again, we used a grid size of 667 nm for the second device.

**Fig. 3:**
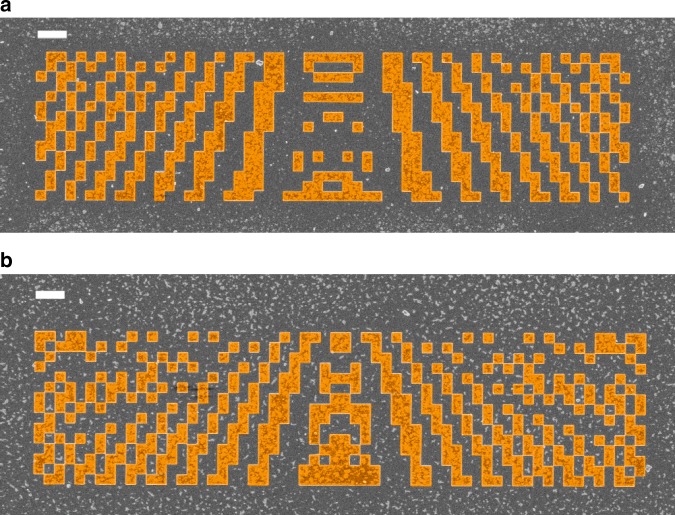
SEM micrographs of the fabricated samples for the two elements on which we concentrate here, and the elements are highlighted in light brown. **a** Element 1 serves the purpose of focusing the BSW 5 µm behind the element. **b** Element 2 serves the purpose of focusing the BSW directly behind the element with a focal width <0.5 λ_BSW_. The spatial extent covered by the element is 40 µm by 10 µm, and each pixel of the checkerboard has a side length of 667 nm. The white scale bar indicates 2 µm. The applied conductive layer to enable SEM imaging on the dielectrics results in visible artifacts as tiny particles, but the layer is removed before the SNOM measurements

The elements are fabricated with standard procedures (see Materials and Methods section). To verify the presence of the designed structure and the quality of the fabrication, Fig. 3 shows scanning electron microscopy (SEM) micrographs of the fabricated elements. The critical features of the structure patterns are well above what can be resolved with our e-beam lithographic system; hence, they are excellently reproduced from the original design. The conspicuous artifacts arise from a conductive anti-charging layer used for SEM imaging on dielectrics, which is removed before the SNOM measurements.

**Fig. 4:**
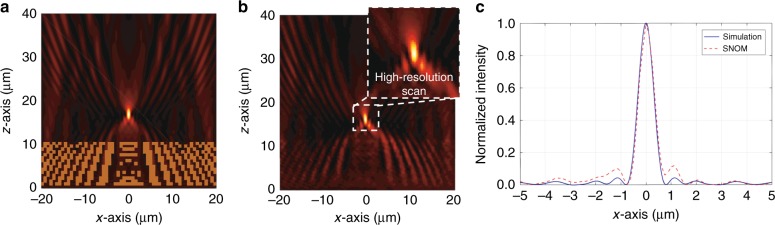
Characterization of a device that focuses BSWs 5 µm behind the functional element. **a** Simulated and **b** measured intensity distributions of the BSW around device 1 (see Fig. 3a) that focuses the incident BSW 5 µm behind the element. The inset in **b** shows the intensity distribution obtained in a high-resolution scan in close proximity to the focal region. **c** Extracted *x*-axis line plots of the intensity through the focus. The intensities are normalized

**Fig. 5:**
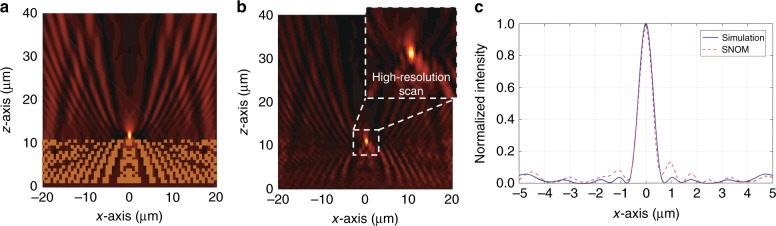
Characterization of a device that focuses BSWs directly behind the functional element. **a** Simulated and **b** measured intensity distribution of the BSW around device 2 (see Fig. 3b) that focuses the incident BSW directly behind the element. The inset in **b** shows the intensity distribution obtained in a high-resolution scan in close proximity to the focal region. **c** Extracted *x*-axis line plots of the intensity through the focus. The intensities are normalized

We use a scanning near-field optical microscope (SNOM) to visualize the interaction of the surface waves with the functional elements. The instrument measures, with the respective tip and the operational wavelength, the evanescent electric field distributions on the top of the PC multilayer stack. Hence, we can compare the measured intensity distribution to that of the simulated electric field intensity, i.e., $\left| {E_{\mathrm{x}}} \right|^2 + \left| {E_{\mathrm{z}}} \right|^2$. We perform two sets of measurements for each device. First, we map the light intensity over a 40 μm × 40 μm area to observe the overall field distribution of the surface wave after interaction with the functional element. The spatial resolution of this scan is 250 nm. In particular, mapping the light field over the entire structure allows us to locate the focal region with respect to the position of the functional element. Afterwards, to precisely measure the focal width, a scan with a resolution as high as 125 nm is performed over a 15 μm × 15 μm area around the focal region, which is cropped to a 5 μm × 5 μm area and displayed as an inset in Figs. 4b and 5b.

Figure 4 shows the simulated and measured intensity data of the first device (see Fig. 3a), i.e., the one with the focus 5 µm behind the element. The simulated and measured intensity distributions are shown in Fig. 4a, b, respectively. The inset in b shows the intensity distribution around the focus in more detail, which is obtained from the high-resolution scan with 125 nm resolution. Figure 4c shows *x*-axis line plots through the focus of both the simulation and experiment to facilitate a quantitative comparison. Excellent agreement is observed between the simulation and the measurement. The only noticeable deviation in the measurement is a small tilt of the intensity pattern that emerges due to a minor alignment error with respect to the SNOM setup, which does not affect the quality of the measurements. Except for this discrepancy, the SNOM measurement demonstrates excellent reproducibility of the simulated result in a quantitative and qualitative manner. First, this justifies the 2D treatment of the simulation. Possible out-of-plane scattering losses at the corrugated surface, which are obviously not taken into account in the 2D simulation scheme yet present in the experiment, are negligible. They do not affect the focusing capability of the fabricated devices. In addition, the simulated and measured focal widths are in fairly good agreement. The focal widths were extracted as 708 nm (simulation) and 718 nm (measurement), which correspond to 0.50*λ*_BSW_ and 0.51*λ*_BSW_, respectively. Such a value is expected for devices that are designed to preserve a certain working distance from the edge of the element. It clearly demonstrates that we can achieve a focal width that hits the lower boundary of what is feasible with far-field optical elements, i.e., in the far field with respect to the functional element along the propagation direction of the surface wave.

To surpass the canonical limit and achieve a focal width that is smaller than half the wavelength of the BSW, we place the focal point directly behind the terminating edge of the functional element. When we do this, it is possible to capitalize on the near-field effect, as the focal point is placed in the proximity of the edge of the functional element along the propagation direction of the BSW. This near-field effect can lead to subwavelength focusing and ultimately allow us to go beyond the canonical limit of 0.5 λ_BSW_. This performance is achieved with the second device. The simulated and measured intensity distributions of the second device with the focal point directly behind the functional element are shown in Fig. 5a and b. Figure 5c shows *x*-axis line plots through the focus of both the simulation and experiment. Again, excellent agreement is found between the simulation and the measurement. Moreover, the focal width in the simulation is 662 nm, which corresponds to a value of 0.47*λ*_BSW_. In the experiment, we even encounter a slightly smaller focal width of 625 nm, corresponding to a value of 0.44*λ*_BSW_. In this way, we experimentally demonstrate that it is possible not only to focus BSWs down to the classical diffraction limit but also to surpass it.

## Discussion

We used a computational approach to design focusing elements for Bloch surface waves that are notoriously challenging to focus due to the limited index contrast. By relying on strategies geared towards the solution of the inverse problem, we defined material distributions on a checkerboard pattern that allowed focusing BSWs at a predefined location. Two-dimensional simulations were performed to optimize the structure pattern of the functional element. Our ideas have been verified in devoted experiments, in which selected devices were fabricated and the intensity distributions of those devices were investigated by means of a scanning near-field optical microscope. Excellent agreement between simulations and experiments was observed. For the device with a focal point farther away from the edge of the functional element, a focal width exactly half the wavelength of the BSW was simulated and verified in the experiment with the fabricated device. For the case of a focal point directly behind the functional element, the focusing of the BSW was predicted to be even below half the wavelength, which was experimentally confirmed. All together, our work lays a solid foundation to control the propagation of BSWs in integrated optical circuits placed on the top surface of a 1D PC multilayer stack. In the design of these circuits, we no longer have to strictly rely on the extrapolation of elements from established macroscopic systems, but we can rely on computational approaches to achieve functional elements, which have also been proven in other technical platforms, such as planar waveguide circuits^1,26–28^. The designed elements can find immediate use, for instance, in lab-on-chip systems where tightly focused BSWs interact with materials carried in fluidic channels to perform spectroscopic measurements.

In addition to the application to BSWs, our work demonstrates how electromagnetic fields can be efficiently controlled when only a limited index contrast is available for steering. This likely applies to many systems that support surface waves, since a large index contrast also frequently implies a large impedance mismatch. This often causes out-of-plane scattering losses that degrade the quality of the device as it limits the propagation length of the surface wave. In our approach, we circumvent the necessity of a large index contrast to control the propagation of the surface waves. Along these lines, we unlock novel opportunities to design integrated optical circuits that rely on surface waves, which is something that is urgently needed in the broader context of applications such as addressing single molecules^29^, photonic quantum devices for fundamental studies^30^, and enhancing sensing capabilities using the quantum nature of light^31^.

## Materials and methods

### Bloch surface wave multilayer platform

We design multilayers for TE polarization at an operation wavelength of 1555 nm. The material stack consists of five double layers on top of a SiO_2_ substrate. Each double layer consists of 260 nm of Si_3_N_4_ and 450 nm of SiO_2_; hence, the terminating layer is SiO_2_. The structured device layer on top of the 1D PC is 70 nm of Si_3_N_4_. Numerical calculations of the dispersion relation of the BSW were performed with a transfer matrix method^11^ and are presented in Sections 1 and 2 of the Supplementary Information.

### Numerical modeling

For the design of the functional elements, we rely on the finite-difference frequency-domain (FDFD) method as implemented in the frequency domain solver of the open source software package Meep^32^. In the simulations, we consider a finite two-dimensional (2D) spatial region that corresponds to the area occupied by the functional element and some space beyond. The space is assumed to be invariant in the third dimension. We consider the space to be filled with a medium according to the spatial distribution of the effective index of the BSW and study the propagation of plane waves with an electric field polarization in the *x*-direction. The electric field then also has a component in the *z*-direction, while the magnetic field only has a component in the invariant *y*-direction, corresponding to the experimental situation. Plane waves are launched into the computational domain that is surrounded by perfectly matched layers. The resulting field distributions are in excellent agreement with full-wave three-dimensional simulations, as reported in the Supplementary Information (see Section 5). Moreover, comparisons to the experiments justify our approach.

### Optimization area

The coordinate system has its origin, i.e., (*x*, *z*) = (0, 0), 1 μm below the lower edge of the functional element and in its center. Therefore, in our particular case, we considered *a*_*x*_ = −0.5 µm and *b*_*x*_ = 0.5 µm as the spatial restriction in the *x*-direction. For the spatial restriction in the *z*-direction, we defined initial values of *a*_*z*_ = 15.5 µm and *b*_*z*_ = 16.5 µm to optimize the functional element to have a focal point 5 µm behind its terminating edge.

### Optimization approach

The optimization procedure is written in Python^33^, using the Python API of Meep^32^ for the FDFD simulations. When each inclusion is modified, the simulations to be performed are distributed over a computing cluster. Running on 100 cores (mixed Intel Xeon X5670 & X5570), this allows the optimization of material grids with half-wavelength feature sizes within as little as one hour, while larger feature sizes can be optimized within a few minutes. We report on further aspects of the optimization routine in the Supplementary Information.

### Fabrication

First, the multilayer stack for the BSW and the device layer is fabricated by plasma-enhanced chemical vapor deposition (PECVD, PlasmaLab 80 Plus by Oxford). Silane, ammonia, and nitrous oxide are used as precursor gases at a process temperature of 300 °C. Second, the spatially structured device layer has been defined on top of the PC multilayer stack. We use e-beam lithography and subsequent reactive-ion etching. We use a negative-tone photoresist (ma-N 2403) and a conductive polymer (Espacer) to avoid charging the sample during the lithographic process. The exposure is performed in a JEOL JBX-5500FS e-beam writer with an accelerating voltage of 50 kV. After removal of the conductive polymer in deionized water and the development (MF-319) of the photoresist, the device layer is etched in CHF_3_ and O_2_ plasma (Oxford RIE 80). The resist is removed in an O_2_ plasma. More details on the fabrication process are provided in the Supplementary Information.

### Optical near-field measurements

We characterize the devices using a scanning near-field optical microscope (SNOM). To excite the BSWs, linearly polarized light at *λ*_0_=1555 nm is first collimated and then illuminated through the prism onto the surface of the BSW multilayer structure (see Fig. 1). The illumination angle is adjusted such that the incident light couples to the BSWs at the phase matching angle, *θ* = 49.5°, inside the glass prism. The evanescent field of the surface wave is collected point by point with a metallized 200-nm-aperture-size SNOM fiber probe, which can map the near-field with high spatial resolution beyond the classical diffraction limit.

## Electronic supplementary material

Supplementary Information

## References

[1] Xie ZW (2018). Ultra-broadband on-chip twisted light emitter for optical communications. Light Sci. Appl..

[2] Mekis A (1996). High transmission through sharp bends in photonic crystal waveguides. Phys. Rev. Lett..

[3] Wu Q, Turpin JP, Werner DH (2012). Integrated photonic systems based on transformation optics enabled gradient index devices. Light Sci. Appl..

[4] Cannon BL (2015). Excitonic AND logic gates on DNA brick nanobreadboards. ACS Photonics.

[5] Wang MJ (2018). Magnetic spin–orbit interaction of light. Light Sci. Appl..

[6] Chen Y, Lin HT, Hu JJ, Li M (2014). Heterogeneously integrated silicon photonics for the mid-infrared and spectroscopic sensing. ACS Nano.

[7] Cadarso VJ, Llobera A, Puyol M, Schift H (2016). Integrated photonic nanofences: combining subwavelength waveguides with an enhanced evanescent field for sensing applications. ACS Nano.

[8] Zhang DG (2017). Silver nanowires for reconfigurable Bloch surface waves. ACS Nano.

[9] Raether, H. *Surface Plasmons on Smooth and Rough Surfaces and on Gratings* (Springer, Berlin, 1988).

[10] Zhang DG (2018). Extending the propagation distance of a silver nanowire plasmonic waveguide with a dielectric multilayer substrate. Nano. Lett..

[11] Yeh P, Yariv A, Hong CS (1977). Electromagnetic propagation in periodic stratified media. I. General theory. J. Opt. Soc. Am..

[12] Dubey R (2017). Experimental investigation of the propagation properties of Bloch surface waves on dielectric multilayer platform. J. Eur. Opt. Soc. Rapid Publ..

[13] Michelotti F (2017). Design rules for combined label-free and fluorescence Bloch surface wave biosensors. Opt. Lett..

[14] Rizzo R (2018). Bloch surface wave enhanced biosensor for the direct detection of Angiopoietin-2 tumor biomarker in human plasma. Biomed. Opt. Express.

[15] Koju V, Robertson WM (2017). Leaky Bloch-like surface waves in the radiation-continuum for sensitivity enhanced biosensors via azimuthal interrogation. Sci. Rep..

[16] Lerario G (2017). High-speed flow of interacting organic polaritons. Light Sci. Appl..

[17] Yu LB (2014). Manipulating Bloch surface waves in 2D: a platform concept-based flat lens. Light Sci. Appl..

[18] Kim MS (2017). Subwavelength focusing of Bloch surface waves. ACS Photonics.

[19] Gao YK, Gan OQ, Xin ZM, Cheng XH, Bartoli FJ (2011). Plasmonic Mach–zehnder interferometer for ultrasensitive on-chip biosensing. ACS Nano.

[20] Zaki AO, Kirah K, Swillam MA (2016). Integrated optical sensor using hybrid plasmonics for lab on chip applications. J. Opt..

[21] Kim MS, Vosoughi Lahijani B, Herzig HP (2018). Stepwise Luneburg lens for Bloch surface waves. Appl. Sci..

[22] Sfez, T. Investigation of surface electromagnetic waves with multi-heterodyne scanning near-field optical microscopy. PhD thesis, École polytechnique fédérale de Lausanne, Lausanne.

[23] Wang RX (2018). Two-dimensional photonic devices based on Bloch surface waves with one-dimensional grooves. Phys. Rev. Appl..

[24] Chen JX, Zhang DG, Wang P, Ming H, Lakowicz JR (2018). Strong polarization transformation of Bloch surface waves. Phys. Rev. Appl..

[25] Miller, O. D. Photonic design: from fundamental solar cell physics to computational inverse design. PhD thesis, University of California, Berkeley.

[26] Piggott AY (2015). Inverse design and demonstration of a compact and broadband on-chip wavelength demultiplexer. Nat. Photonics.

[27] Yu ZJ, Cui HR, Sun XK (2017). Genetically optimized on-chip wideband ultracompact reflectors and Fabry–Perot cavities. Photonics Res..

[28] Yu ZJ, Cui HR, Sun XK (2017). Genetic-algorithm-optimized wideband on-chip polarization rotator with an ultrasmall footprint. Opt. Lett..

[29] Yang H, Cornaglia M, Gijs MAM (2015). Photonic nanojet array for fast detection of single nanoparticles in a flow. Nano. Lett..

[30] Vest B (2017). Anti-coalescence of bosons on a lossy beam splitter. Science.

[31] Lee C (2016). Quantum plasmonic sensing: beyond the shot-noise and diffraction limit. ACS Photonics.

[32] Oskooi AF (2010). M_EEP_: a flexible free-software package for electromagnetic simulations by the FDTD method. Comput. Phys. Commun..

[33] Python Software Foundation. Python language reference, version 3.4. Available at http://www.python.org.

