# Supplementary material for: Inverse photonic design of functional elements that focus Bloch surface waves
Source: Light Sci Appl. 2018 Dec 12;7:104. doi: 10.1038/s41377-018-0106-x (PMC6289961; doi:10.1038/s41377-018-0106-x)
Supplement: Supplementary file 1 — Supplementary Information [file 41377_2018_106_MOESM1_ESM.docx]

Inverse Photonic Design of Functional Elements that Focus Bloch Surface Waves

Yannick Augenstein^1,*^, Andreas Vetter^2,3,*^, Babak Vosoughi Lahijani^4^, Hans Peter Herzig^4^, Carsten Rockstuhl^1,2,**^, and Myun-Sik Kim^4,**^

^1^ Institute of Theoretical Solid State Physics, Karlsruhe Institute of Technology, 76131 Karlsruhe, Germany

^2^ Institute of Nanotechnology, Karlsruhe Institute of Technology, 76344 Eggenstein-Leopoldshafen, Germany

^3^ SUSS MicroOptics SA, Rogues-Terres 61, Hauterive, CH 2068, Switzerland

^4^ Optics & Photonics Technology Laboratory, Ecole Polytechnique Fédérale de Lausanne (EPFL), Neuchâtel, Switzerland

^*^Authors have equally contributed as the first author.

^**^ Corresponding authors: Carsten Rockstuhl (carsten.rockstuhl@kit.edu), Myun-Sik Kim (myunsik@hotmail.com)

# Table of Contents

[1. Design of the Bloch surface wave platform 3](#_Toc530403510)

[2. Wavelength-dependence of the effective refractive index 4](#_Toc530403511)

[3. Fabrication of the Bloch surface wave plattform 5](#_Toc530403512)

[4. Fabrication of the functional element 5](#_Toc530403513)

[5. Comparison of 2D and 3D simulations 6](#_Toc530403514)

[6. Convergence properties of optimization 9](#_Toc530403515)

[7. Sensitivity of the device to the illumination profile 10](#_Toc530403516)

[8. Analysis of lateral and axial focal width 12](#_Toc530403517)

[9. Broadband response of the device 14](#_Toc530403518)

[10. References 15](#_Toc530403519)

# Design of the Bloch surface wave platform

The multilayer platform for the 1D photonic crystal is designed by calculating the band-gap diagram and dispersion curves using a transfer matrix method.^1^ The designed multilayer stack consist of five periods of silicon nitride (Si_3_N_4_, $n_{\mathrm{Si}_{3}N_{4}}$= 1.94, *t* = 260 nm) and silicon dioxide (SiO_2_, $n_{\mathrm{Si}O_{2}}$ = 1.47, *t* = 450 nm), with *t* being the thickness. For the device layer, which forms the checkerboard element by spatial patterning, an additional 70 nm of Si_3_N_4_ is deposited on top of the PC. Figure S1 shows the band-gap diagram and dispersion curves without and with the device layer, which leads to the index contrast of 0.1.


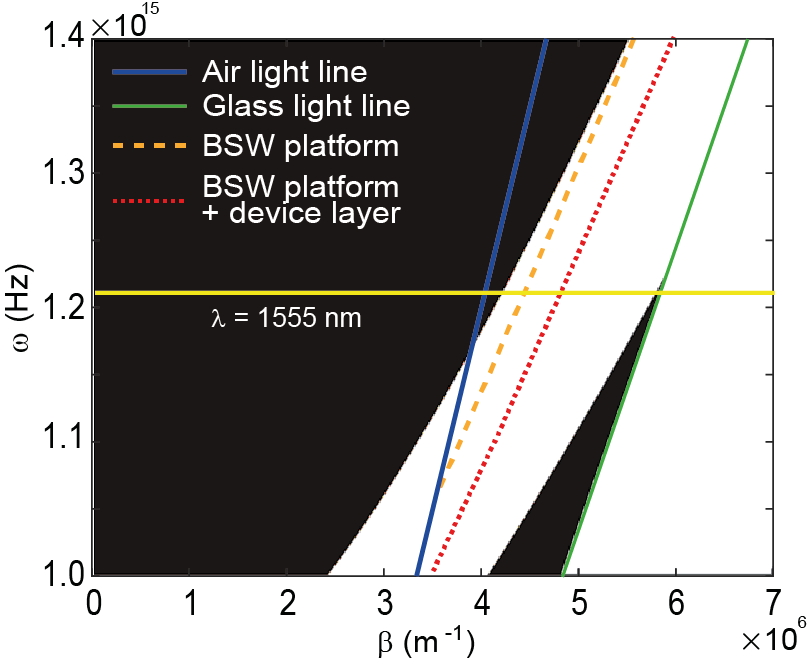


***Figure S1.*** *Dispersion curves of the designed multilayer platform without and with the device layer (70 nm of Si_3_N_4_) and the band-gap diagram along with the air light line and the glass light line. The clear area is the localized photonic band-gap.*

# Wavelength-dependence of the effective refractive index

In order to assess the performance of the devices over a broad wavelength range, we simulate the effective refractive indices of the device platform, following the procedure described in the previous section. The materials SiO_2_ and Si_3_N_4_ are assumed to be non-dispersive over the wavelength regime under consideration. Figure S2 depicts the effective refractive indices in a wavelength range of 1300 to 1650 nm, together with the difference in the refractive index.


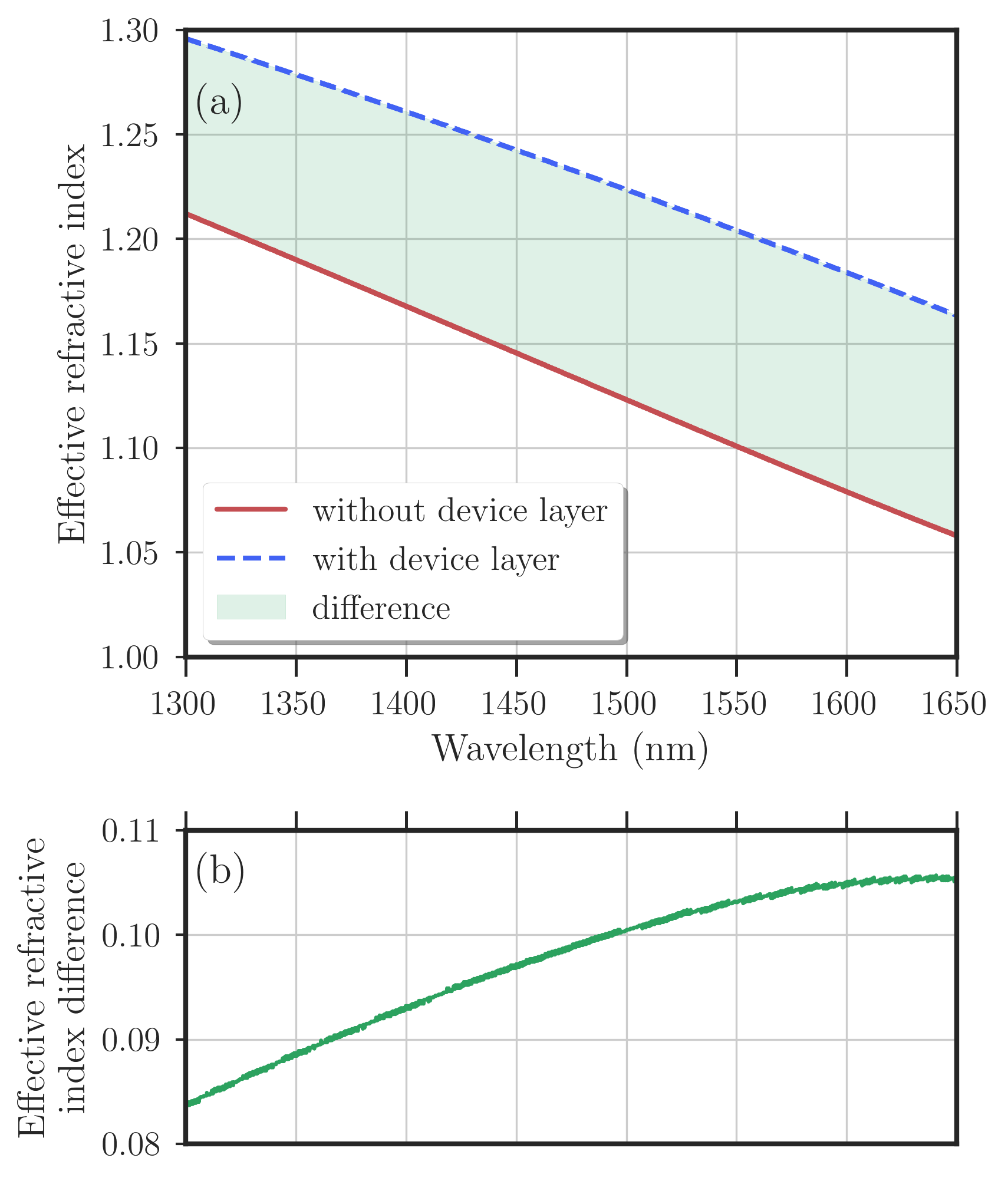


***Figure S2.*** *(a) Effective refractive index for the device platform under consideration, without (drawn through red) and with device layer (dashed blue). The difference in the effective refractive index is indicated in green. (b) Effective refractive index difference.*

# Fabrication of the Bloch surface wave plattform

For the multilayer stack of the one-dimensional PC, five periods of 260 nm of Si_3_N_4_ and 450 nm of SiO_2_ are alternately deposited on a fused silica substrate by using plasma-enhanced chemical vapor deposition (PECVD, PlasmaLab 80 Plus by Oxford), and it is terminated by SiO_2_. Employed precursor gases are silane (SiH_4_), ammonia (NH_3_), and nitrous oxide (N_2_O), and the process temperature is 300 °C. For the device layer, 70 nm of Si_3_N_4_ is deposited on top of the PC stack.

# Fabrication of the functional element

To structure the device layer, we rely on e-beam lithography and reactive-ion etching. First, the chip containing the multilayer stack is cleaned in an acetone ultrasonic bath and rinsed in isopropanol. Organic residues are removed in an oxygen plasma cleaner.

For adhesion promotion, we apply TI Prime (MicroChemicals) and bake the chip on a hotplate for 3 min at 100 °C. Subsequently, the negative tone resist ma-N 2403 (micro resist) is spin-coated. After the prebake on a hotplate at 90 °C for 2 min, the resist thickness is around 340 nm. To avoid charging of the non-conducting substrate during e-beam lithography, we apply a thin layer of conductive polymer (Espacer 300Z, Showa Denko K.K.).

As a third step, e-beam lithography is performed on a JEOL JBX-5500FS machine with an accelerating voltage of 50 kV and a step size of 4 nm. The exposure dose amounts to 200 µC·cm^−2^ at a writing current of 100 pA.

After the exposure, the conductive layer is removed in a deionized water bath. Subsequently, the sample is developed in MF-319 (MicroChem) for 1 min and rinsed in deionized water. The patterns in the negative resist are transferred to the device layer by using reactive ion etching (Oxford RIE 80), CHF_3_ and O_2_ plasma for 75 s. Finally, the remaining resist is removed in an O_2_ plasma. For SEM imaging, again a conductive film (Espacer) is applied, and removed afterwards in a deionized water bath.

# Comparison of 2D and 3D simulations

In order to justify our optimization approach relying on 2D simulations in Meep, we reproduce the results relying on full-wave 3D simulation. Due to the complexity of the simulation, we use a time domain solver (CST Microwave® Studio 2018). The mesh resolution is set to ten cells per wavelength. The boundary conditions are introduced as perfectly matched layers (PMLs). We use the same geometry and refractive indices introduced in the first section of the Supplementary Information. Please note, no effective index or something similar is considered; but just the actual geometry. In these simulations, we excite the guided mode of the structure. An enlarged cross-sectional area is used as the input port. The polarization is set to the TE mode (along the x-direction), at an excitation wavelength of 1555 nm. After running the simulation, the electric field is retrieved directly above the device layer, as well as some cuts along the structure (see Figure S3 for the results of the device with a feature size of 667 nm). Furthermore, we show the field plots of the full-wave 3D simulations in Figure S4a-d and compare the intensity profiles in the focal plane to the 2D simulations in Figure S4e-h for the different feature sizes considered in Figure 2 of the manuscript. The excellent agreement between our full-wave 3D simulations and the 2D simulations confirm the validity of our 2D optimization approach.

The 3D simulations are performed on one node of the *bwunicluster* (16 cores, 110 GB memory, runtime of 8 h per simulation).

*
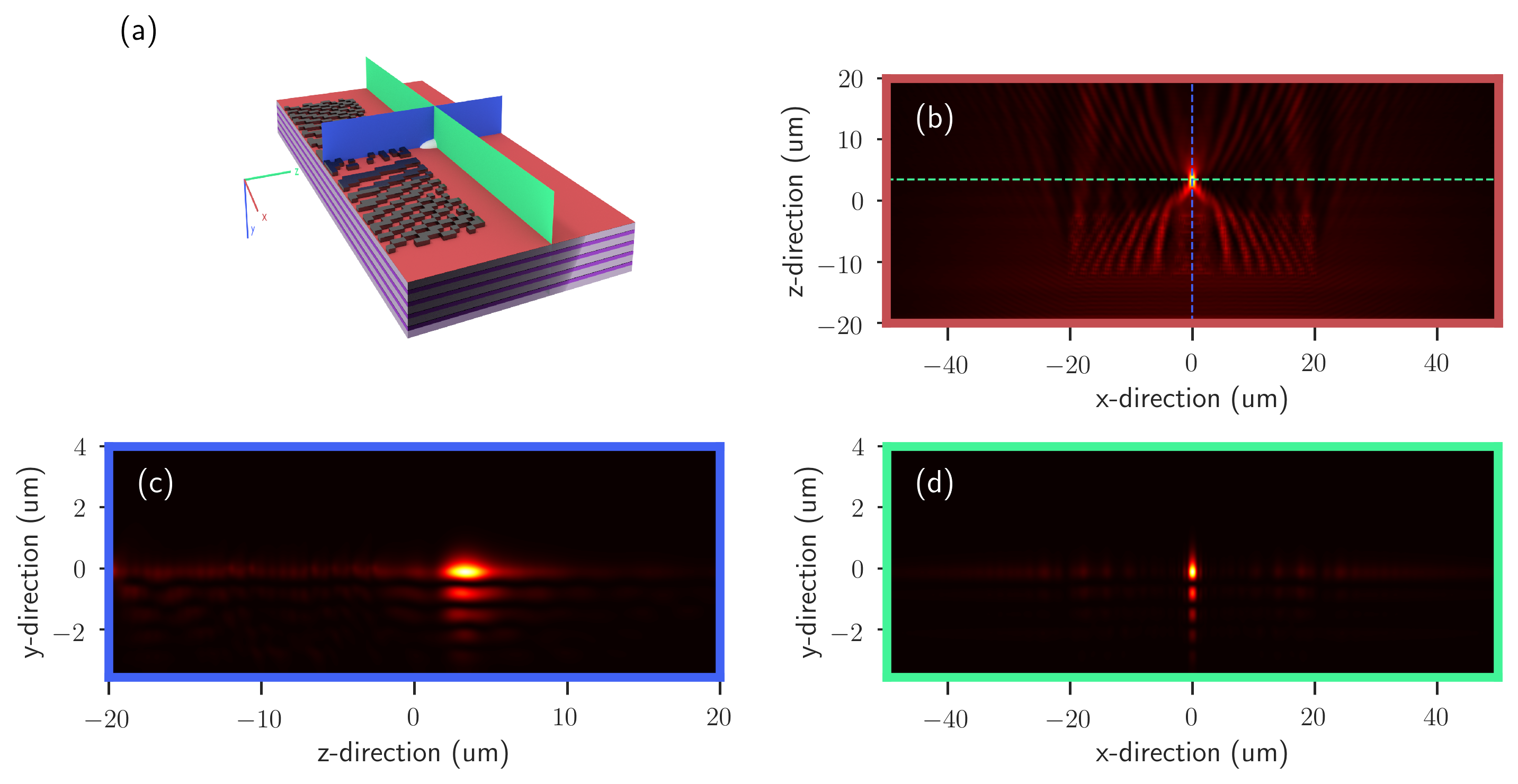
*

***Figure S3.*** *3D simulation results. (a) Sketch of the 3D simulation setup. We retrieve the 3D electric fields in three planes, indicated by different colors. This color corresponds to the frame color in (b), (c), and (d). The focal volume is indicated by a white ellipsoid. (b) Total electric field intensity in the x-z plane, directly on top of the device layer. (c) Total electric field intensity in the y-z plane in the middle of the device, along the propagation direction. (d) Total electric field intensity in the x-y plane (the focal plane).*


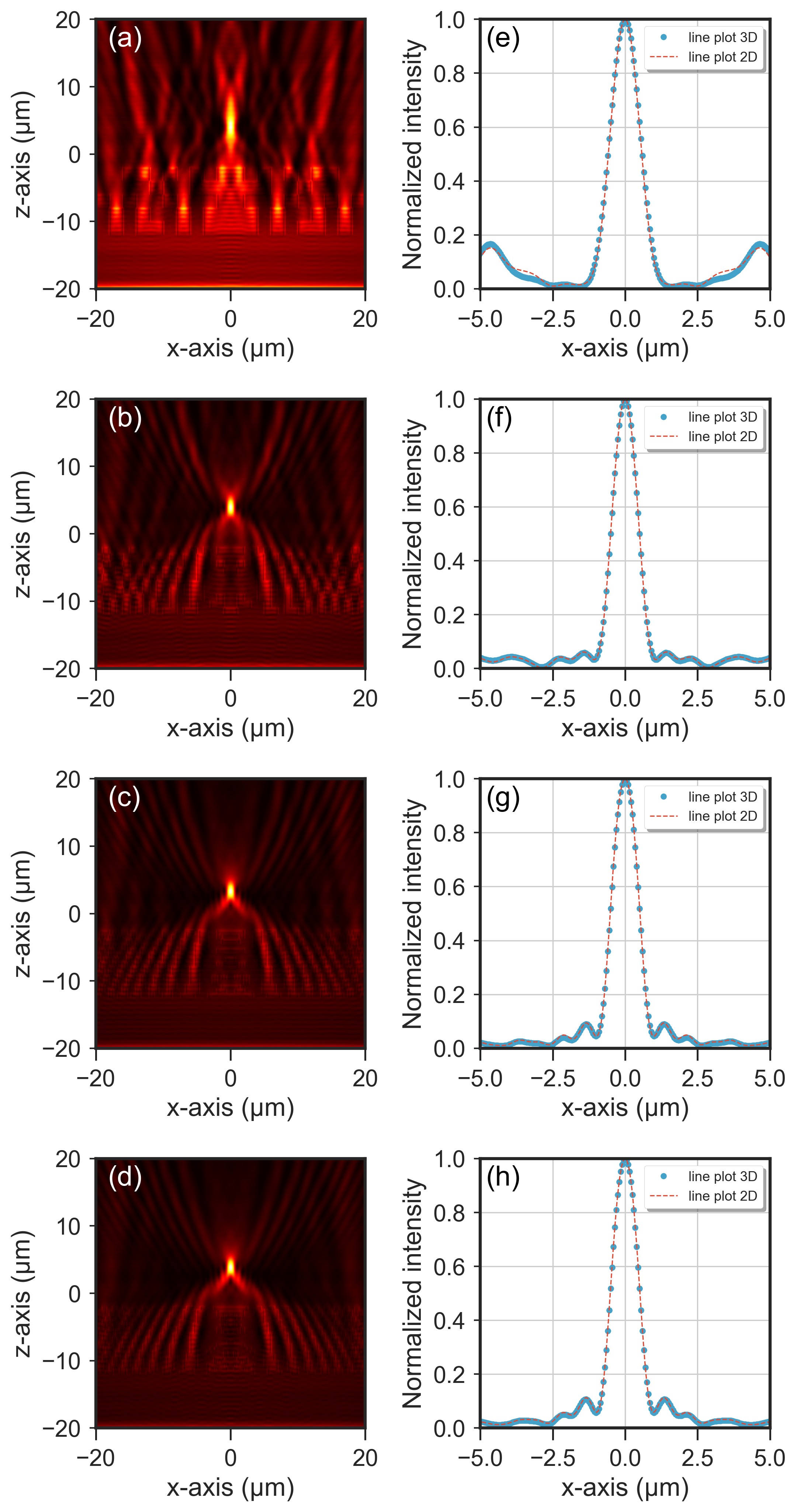


***Figure S4.*** *Comparison of 2D and 3D simulation results. (a)-(d) Total electric field intensity in the x-z plane, as retrieved from 3D simulations, for devices with different feature sizes [(a) 2000 nm, (b) 1000 nm, (c) 667 nm, and (d) 500 nm]. (e) - (h) Comparison of line plots along the x-axis between 2D (dashed red line) and 3D (blue circles) simulations.*

# Convergence properties of optimization

Figure S5a shows the figure of merit (FOM) evolution of our optimization algorithm at different feature sizes. Notably, optimizations for larger feature sizes converge much faster than those of smaller feature sizes, but the total FOM (intensity in focus) improves with smaller feature sizes. This agrees with the data shown in Figure 2 of the manuscript. Figure S5b shows that the number of iterations needed for convergence to a certain FOM grows exponentially with the number of design parameters of the device.


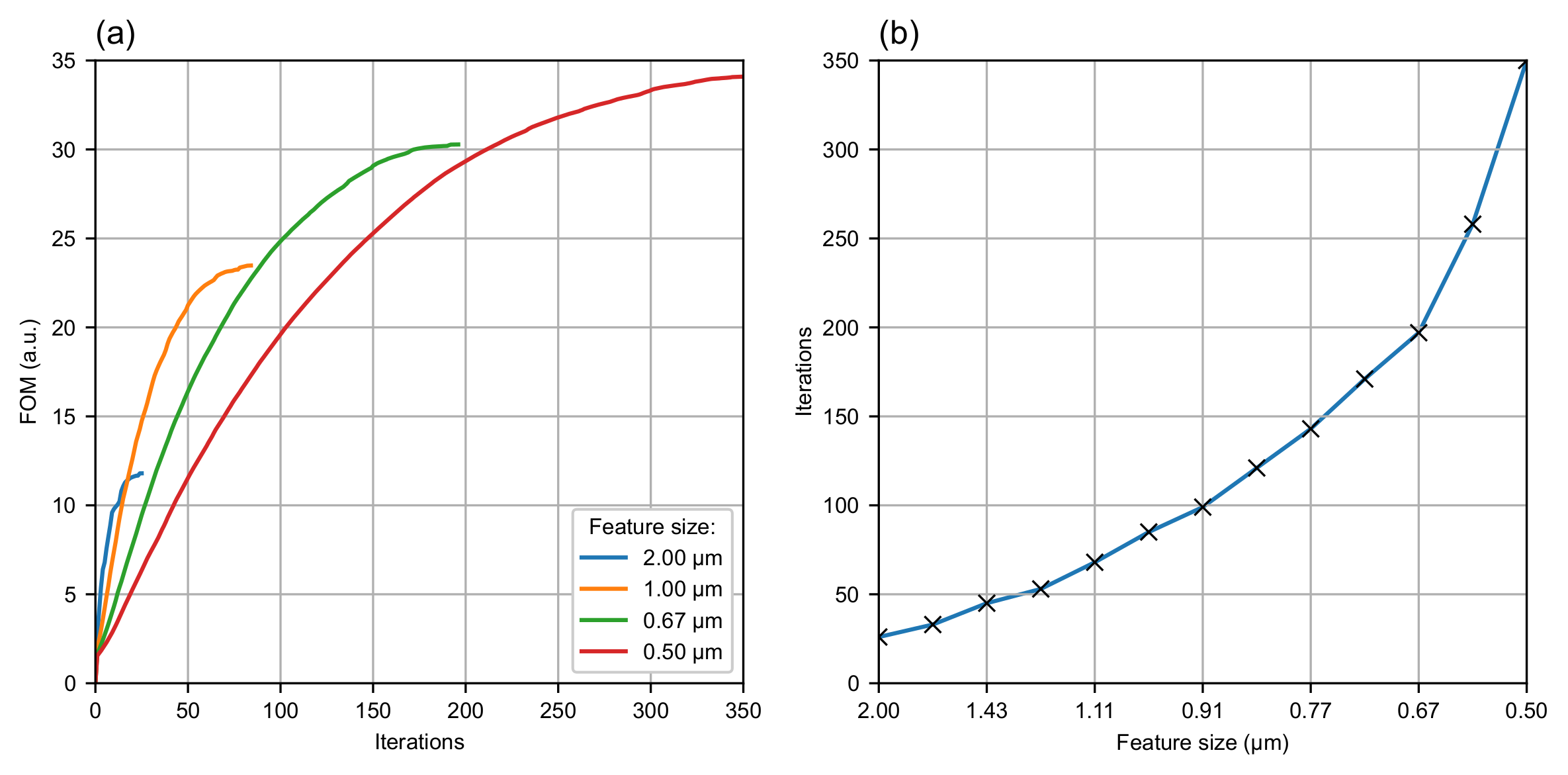


***Figure S5.*** *(a) FOM evolution as a function of the number of iterations, for different feature sizes (compare Figure 2 in the manuscript). (b) Number of iterations for optimizations of different feature sizes, until a certain FOM is reached. The feature sizes correspond to the optimizations shown in Figure 2a-d in the manuscript.*

# Sensitivity of the device to the illumination profile

In Figure S6a-d, we show the field profiles resulting from illuminating the device with a feature size of 667 nm with a Gaussian beam of different beam waist diameters. The waist of the Gaussian beam is in a plane shortly before the actual device. This analysis shall simulate the impact of non-perfect plane wave excitation in the experiment. It is apparent that with increasing beam diameter, the intensity and width of the focal point approach our plane wave simulation results. The focal width is within 15% of the classical diffraction limit of 0.5 $\lambda_{\mathrm{BSW}}$ up to a beam diameter of 10 µm (high to low), at which point it starts to widen significantly. The intensity is not normalized to the amplitude incident on the device, but to the total amplitude of the Gaussian beam.

In Figure S7 we also considered the impact of a tilted plane wave incidence onto the device with a feature size of 667 nm. The width of the focal point increases only slightly with a tilt of up to 2°, whereas the intensity drops off slightly faster. At higher angles, the intensity in the focus drops off sharply and the focus shifts up to 3 µm from the origin at 10°. Combining the insights from Figure S6 and S7, we can confidently say that our device is robust against variations in illumination properties.


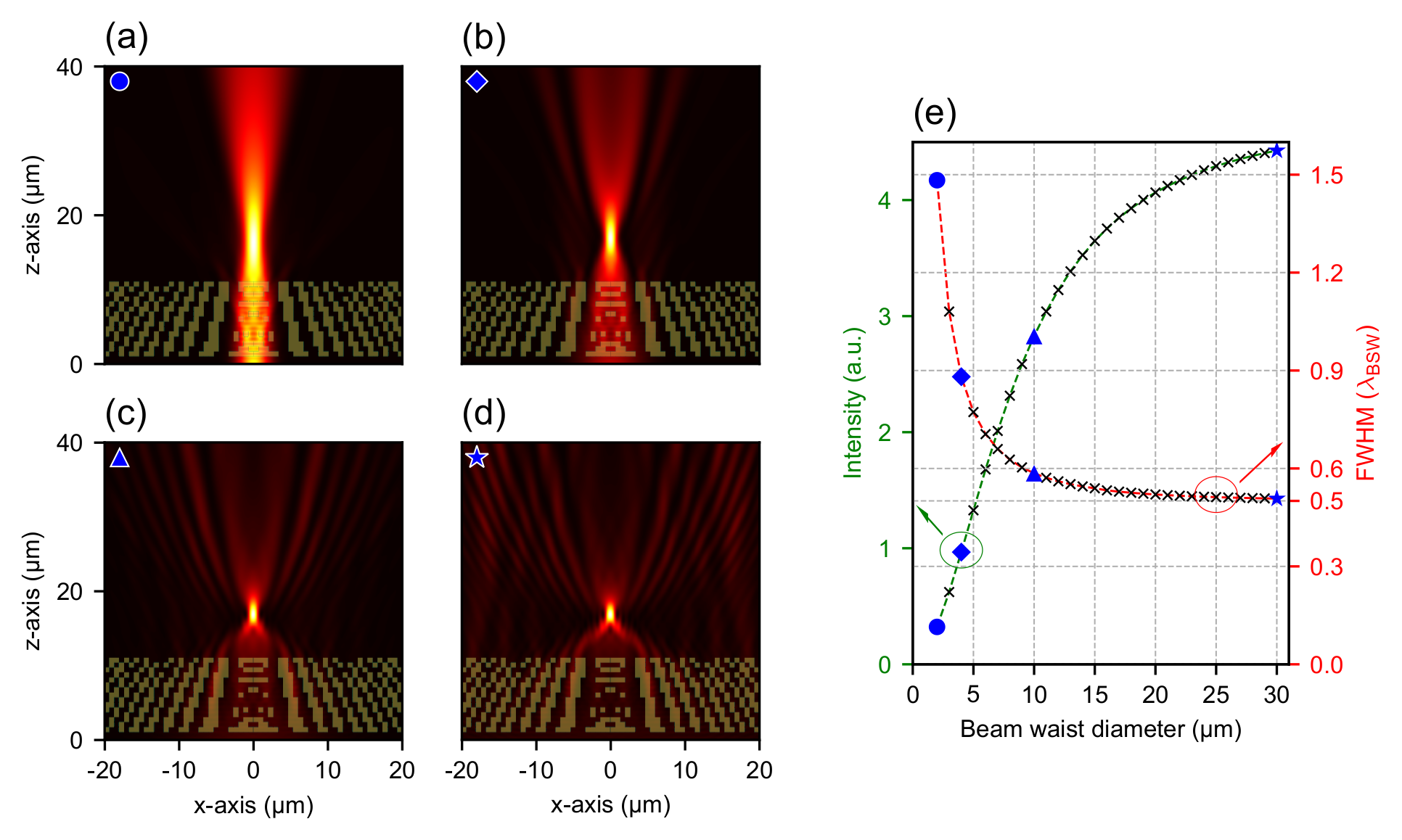


***Figure S6.*** *Gaussian beam with different beam waist diameters illuminating the device with a feature size of 667 nm and the focus 5 μm behind the device. On the left (a) - (d), we show the electric field intensity distributions upon illuminating the element with a Gaussian beam for some selected beam waists. The device with a feature size of 667 nm is shown as an overlay. The considered beam waists correspond to (a) 2 μm, (b) 4 μm, (c) 20 μm, and (d) 30 μm, respectively. (e) We show the achievable focal width and the peak intensity in the focal point of the elements, presented as a function of beam waist diameters. Selected data points correspond to the samples shown in (a) - (d), as marked by the symbol in the top left corner of each intensity distribution.*


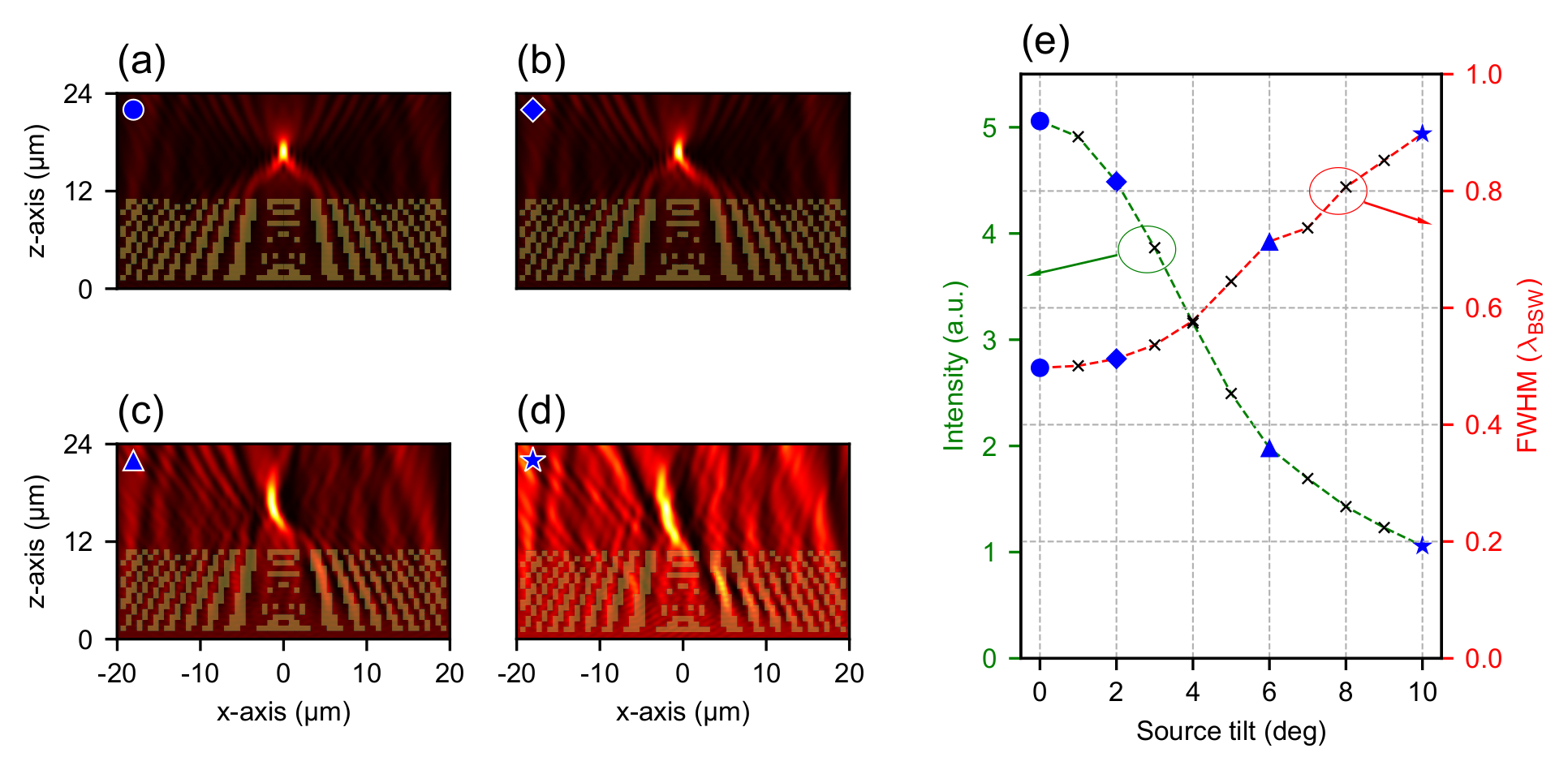


***Figure S7.*** *Plane waves illuminating the device with a feature size of 667 nm and a focus of 5 μm at different incident angles. On the left (a) - (d), we show the electric field intensity distributions upon illuminating the element with a tilted plane wave source. The device is shown as an overlay. The considered tilt angles correspond to (a) normal incidence, (b) 2°, (c) 6°, and (d) 20°, respectively. (e) We show the achievable focal width and the peak intensity in the focal point of the elements shown as a function of source tilt. Selected data points correspond to the samples shown in (a) - (d), as marked by the symbol in the top left corner of each intensity distribution.*

# Analysis of lateral and axial focal width

In Figure 2 of the manuscript, we discuss the focal width of our devices along the lateral x-direction. Here in Figure S8, we additionally introduce a comparison with the axial focal width (along propagation direction) by means of 2D simulations. The axial focal width of the device with a feature size of 667 nm (see Figure S8c) has been extracted to be 2.45 µm, corresponding to 1.73 $\lambda_{\mathrm{BSW}}$. Due to effects of the intermediate field, this surpasses the canonical limit of 2 $\lambda_{\mathrm{BSW}}$ as expected from classical resolution theory for a numerical aperture of one. In general, both axial and lateral FWHMs increase with larger feature sizes.


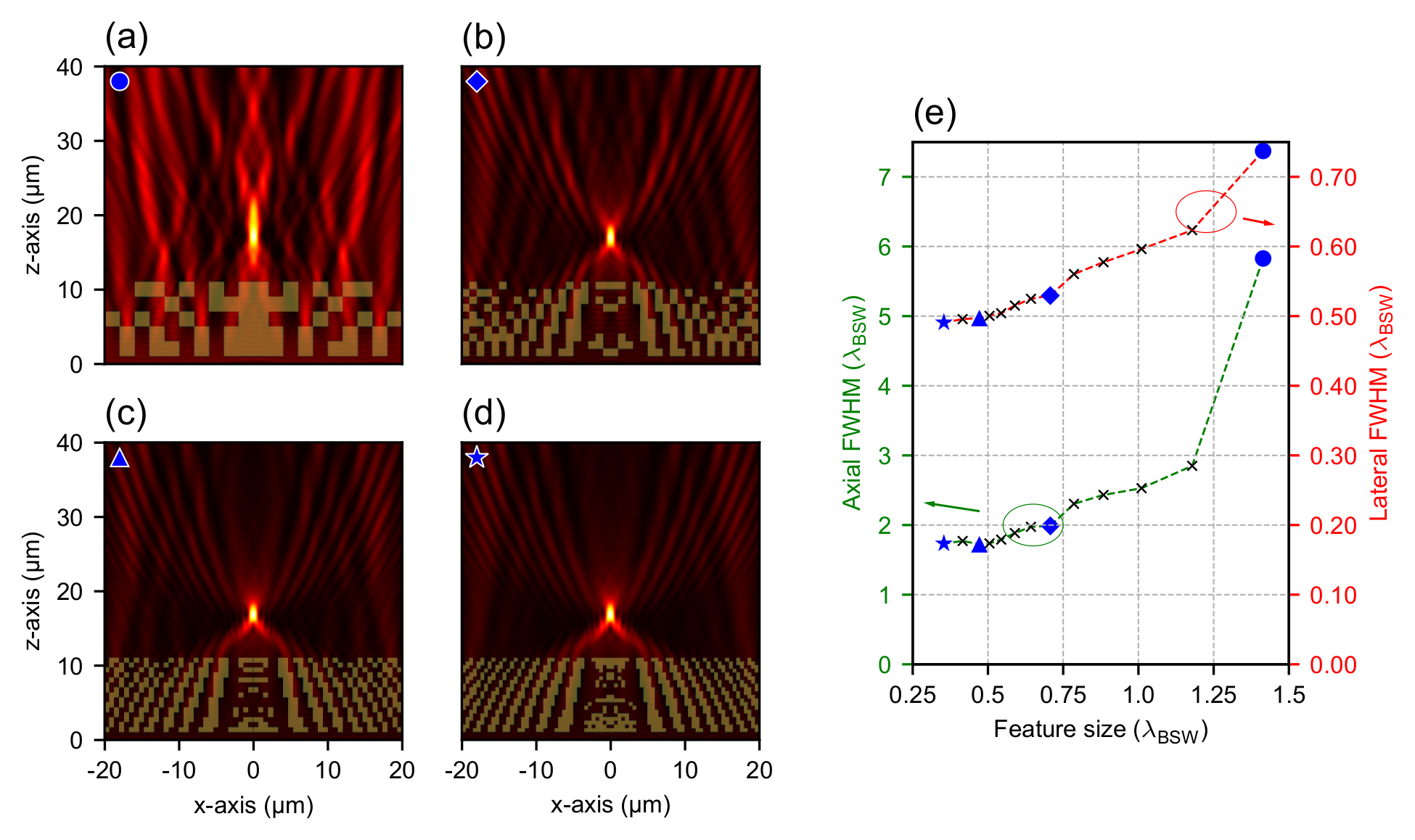


***Figure S8.*** *Optimization of different structures that can focus BSWs at the some distance behind the functional element,* i.e.*, 5 µm in the present case. On the left (a) - (d), we show the electric field intensity distributions upon illuminating optimized elements with a plane wave for some selected spatial discretization of the structures. Please note, these figures are identical to those shown in the main manuscript in Figure 2. They are merely repeated here for convenience. The structures that were optimized are shown as an overlay. The considered grid size corresponds to (a) 2 μm (=1.414 λ_BSW_), (b) 1 μm (=0.707 λ_BSW_), (c) 667 nm (=0.471 λ_BSW_), and (d) 500 nm (=0.354 λ_BSW_), respectively. (e) We show the lateral and axial FWHMs of the focal point as a function of the feature size. Selected data points correspond to the samples shown in (a)-(d), as marked by the symbol in the top left corner of each intensity distribution.*

# Broadband response of the device

To demonstrate the broadband properties of our designs, we simulate the response of the device with a feature size of 667 nm in a wavelength range of 1300 – 1660 nm with a 10 nm step, relying on the previously introduced 2D simulation approach. The results are shown in Figure S9. The variation in the effective index of the device with and without element layer, as presented in the third section of this Supplementary Information, is fully taken into account. We note that the device is optimized to operate at a wavelength of 1550 nm.

Notably, the device achieves a focal width of $0.50\lambda_{BSW}$ and below in the range of 1550 – 1650 nm, demonstrating excellent broadband properties in this range, and still decent properties down to a wavelength of 1400 nm, where the focal width and intensity of the focal spot begin to worsen significantly. The change in the intensity with the wavelength is directly connected to the variation in the effective refractive index difference (compare with Figure S2). It is important to note that the devices can be optimized for a different wavelength, changing the dependence on the wavelength.


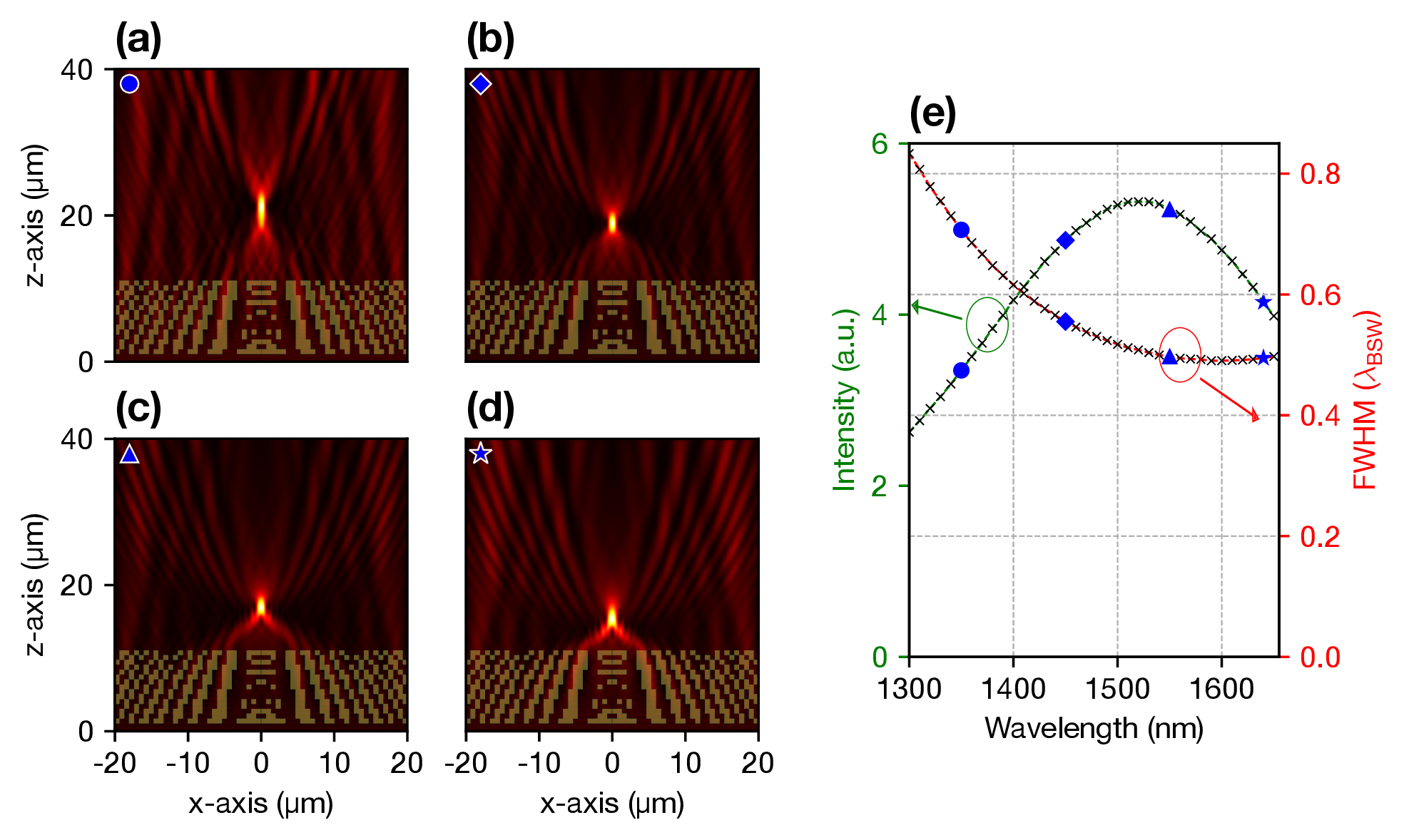


***Figure S9.*** *Plane waves illuminating the device with a feature size of 667 nm and a focus of 5 μm at different wavelengths. On the left (a) - (d), we show the electric field intensity distributions upon illuminating the element with a tilted plane wave source. The 667 nm device is shown as an overlay. The considered wavelengths are (a) 1350 nm, (b) 1450 nm, (c) 1550 nm, and (d) 1650 nm, respectively. (e) The focal width and the peak intensity at the focal point of the elements are shown as a function of wavelength. Selected data points correspond to the samples shown in (a)-(d), as marked by the symbol in the top left corner of each intensity distribution.*

# References

1. Yeh PO, Yariv AM, Hong CS. Electromagnetic propagation in periodic stratified media. I. General theory. *J. Opt. Soc. Am.* **1977**, 67, 423-438.
